# Genome-wide analysis of the CrRLK1L gene family in *Puccinellia tenuiflora* and functional study of PutFER1 in Arabidopsis underpinning salt tolerance

**DOI:** 10.3389/fpls.2025.1680452

**Published:** 2025-11-26

**Authors:** Gaojian Li, Yulin Peng, Yanshuang Liu, Zhen Wu, Shaojun Dai, Zhi Qin, Meihong Sun

**Affiliations:** Development Center of Plant Germplasm Resources, College of Life Sciences, Shanghai Normal University, Shanghai, China

**Keywords:** CrRLK1L gene family, expression profiling, PutFER1, *Puccinellia tenuiflora*, salt stress

## Abstract

CrRLK1L receptor-like kinases have attracted significant interest in plant biology owing to their pivotal roles in development, stress response, and signal transduction. However, our understanding of their importance in the extreme saline-alkali tolerant mechanisms of the halophyte alkaligrass (*Puccinellia tenuiflora*) remains limited. In this work, we analyzed the homology and evolutionary relationships of the CrRLK1L gene family across 30 species. Through genome-wide analysis we identified 25 *PutCrRLK1L* genes, which revealed conserved malectin-like and kinase domain, unique intronless structures, and stress-/hormone-responsive promoter elements. Transcriptomic and RT-qPCR analyses revealed salinity response of nine *PutCrRLK1L* genes’ expression in roots and leaves. Functional characterization of *FER* and antioxidant enzyme activities demonstrated that *PutFER1*-overexpressing Arabidopsis lines exhibited enhanced root growth under salt stress and reduced ROS accumulation compared to wild-type plants. These findings may open new avenues for research into halophyte-specific salt tolerance mechanisms and provide potential candidate genes for improving crop resilience to saline-alkaline soils.

## Introduction

The CrRLK1L (*Catharanthus roseus* RECEPTOR-LIKE KINASE 1-LIKEs) family is named after its first member, *CrRLK1*, isolated from Madagascar periwinkle (Schulze-Muth et al., 1996). These proteins have long been recognized as key regulators of various developmental processes (e.g., cell elongation, polarized growth, and fertilization) and abiotic stresses (e.g., salt stress, extreme temperature, and nutritional deficiency) (Lindner et al., 2012; Nissen et al., 2016; Franck et al., 2018a; Zhu et al., 2021; Zhang et al., 2025). The CrRLK1L family proteins are characterized by two tandemly-linked malectin-like domains (MLDs) required for binding to ligands and cell wall polymers (such as pectin), a transmembrane domain (TMD), and an intracellular kinase domain that relays apoplastic signals to intracellular components via phosphorylation (Schallus et al., 2008; Boisson-Dernier et al., 2011; Kessler et al., 2014). In Arabidopsis, there are 17 CrRLK1Ls, among which the biological functions of FERONIA (FER), THESEUS1 (THE1), HERCULES1 (HERK1), ANXUR1/2 (ANX1/2), ANJEA (ANJ), ERULUS (ERU) and BUDDHA’S PAPER SEAL1/2 (BUPS1/2) have been well addressed (Guo et al., 2009; Boisson-Dernier et al., 2015; Schoenaers et al., 2018; Galindo-Trigo et al., 2019; Ge et al., 2019; Liu et al., 2021a; Cheung, 2024). They regulate diverse processes including cell wall/membrane integrity (e.g., FER, HERK1, and THE1), pollen-ovule interactions (e.g., ANJ and HERK1), pollen tube development (e.g., FER, ANJ, HERK1, ANX1/2, BUPS1/2, and ERU), cell elongation (e.g., THE1 and HERK1), and trichome/tapetum morphogenesis (i.e., CVY1) (Nasrallah et al., 2013; Gachomo et al., 2014; Kessler et al., 2014; Richter et al., 2017; Schoenaers et al., 2018; Zhong et al., 2022). Moreover, some members involved in immune and abiotic stress responses. For instance, FER and THE1 play crucial roles in maintaining cell wall integrity and plasma membrane homeostasis (Richter et al., 2017), while the LETUM1/2 (LET1/2)-LRE-like GPI-AP1 (LLG1) complex participates in disease resistance by activating the SUPPRESSOR of *mkk1 mkk2* (SUMM2)-mediated immune pathway (Huang et al., 2020a). Additionally, MEDOS (MDS) 1–4 are involved in metal ion-regulated developmental processes (Richter et al., 2018). However, their functions in salinity tolerance are largely unknown.

FER, originally described as a regulator of female fertility, is a critical modulator of pollen tube-ovule interaction (Huck et al., 2003; Rotman et al., 2003). Arabidopsis FER is universally expressed in both vegetative and reproductive organs and is the most extensively studied and best-characterized CrRLK1L family protein (Escobar-Restrepo et al., 2007; Franck et al., 2018b; Cheung, 2024). In Arabidopsis, FER regulates the initiation and elongation of plant root hairs by regulating the homeostasis of reactive oxygen species (ROS) (Duan et al., 2010; Yu et al., 2012; Huang et al., 2013). In addition, FER also regulates seed germination, plant cell expansion, cell polar growth, pathogen defense, and abiotic stress tolerance by modulating the homeostasis of phytohormone such as jasmonic acid (JA), salicylic acid (SA), abscisic acid (ABA), brassinosteroid (BR), and ethylene (Guo et al., 2009; Deslauriers and Larsen, 2010; Guo et al., 2018; Zhao et al., 2021; Wang et al., 2024b; Chaudhary et al., 2025; Fu et al., 2025).

Importantly, FER regulates several salt-responsive processes to maintain cellular homeostasis such as activation of pectin methyl esterase (PME), mitogen-activated protein kinase (MAPK) cascade, and intracellular Ca^2+^ signal transduction (Feng et al., 2018; Gigli-Bisceglia et al., 2022). Under salt stress, disassociated RAPID ALKALINIZATION FACTOR (RALF) peptides RALF22/23 bind to FER, inducing its internalization to activate stress responses (Zhao et al., 2018). It has been reported that FER coordinates salt stress responses by modulating stomatal dynamics via G protein β subunit (AGB1) interaction to limit ion influx, phosphorylating serine hydroxymethyltransferase1 (SHM1) to enhance photorespiration for toxicity mitigation, and inactivating phytochrome B (phyB) phosphorylation to inhibit PIF-mediated growth (Yu and Assmann, 2018; Yu et al., 2018; Liu et al., 2023; Jiang et al., 2024a; Liu et al., 2024). Similar to *fer-4* mutation, *herk1 the1–4* mutation also leads to leaf bleaching phenotype under salt stress suggesting that other CrRLK1L family proteins are required for salt tolerance in plants (Gigli-Bisceglia et al., 2022).

Additionally, genome-wide analyses of different species identified 852 members of the CrRLK1L gene families (Zhang et al., 2016; Yang et al., 2020; Rao et al., 2021; Tang et al., 2022; Ma et al., 2023a; Qiao et al., 2023). Among them, 45 in soybean (*Glycine max*), 48 in tobacco (*Nicotiana tabacum*), 24 in maize (*Zea mays*), 43 in wheat (*Triticum aestivum*), and 26 in quinoa (*Chenopodium quinoa*) play important roles under salt stress (Wang et al., 2021; Jiang et al., 2022; Li et al., 2022; Gawande and Sankaranarayanan, 2024; Jiang et al., 2024b; Wang et al., 2024a). Representatively, the highly salinity-induced mRNA levels for soybean *GmCrRLK1L2/20*, tobacco *NtCrRLK1L47*, maize *ZmCrRLK1L1/2*, wheat *TaCrRLK1L1*, and quinoa *CqCrRLK1L5/7/9* indicate that *CrRLK1L* members in these species could participate in salt response (Wang et al., 2021; Jiang et al., 2022; Li et al., 2022; Gawande and Sankaranarayanan, 2024; Jiang et al., 2024b; Wang et al., 2024a).

Alkaligrass (*Puccinellia tenuiflora*) is a monocotyledonous halophyte widely distributed in the saline-alkaline land in North-eastern China. This species can survive under extreme saline-alkaline (600 mM NaCl and 150 mM Na_2_CO_3_) as well as drought and chilling stresses (Zhang et al., 2013; Meng et al., 2016). Due to its high nutritional value and stress tolerance property, alkaligrass serves as a model organism for studying alkali tolerance mechanisms. Recently, the high-quality chromosome-level whole-genome sequencing has revealed that the genome size of alkaligrass is approximately 1.5 Gb and contains 38,387 protein-encoding genes (Zhang et al., 2020). In addition, stress-treated transcriptome profiling uncovers a series of unique saline- and alkaline-responsive genes and a large number of genes in FER and FLS2 signaling pathways were induced by salinity and alkali in roots of alkaligrass (Zhang et al., 2020). Quantitative proteomic studies have revealed stress-induced changes in protein abundance and post-translational modifications (PTMs) under diverse conditions, such as salt stress (NaCl, Na_2_CO_3_, and NaHCO_3_), low temperature, and oxidative stress (Yu et al., 2011, 2013; Meng et al., 2016; Zhao et al., 2016; Yin et al., 2019; Zhang et al., 2019; Suo et al., 2020). Analyses of 25 glutaredoxins (*PutGRXs*) and 54 germin-like protein (*PutGLPs*) genes indicate that *PutGRXs* and *PutGLPs* play critical roles in the saline-alkaline stress response of *P. tenuiflora* (Li et al., 2025; Liu et al., 2025). However, the molecular mechanisms by which the CrRLK1L family members in alkaligrass participates in salt stress responses remain poorly understood. Identification of *PutCrRLK1Ls* required for salt tolerance would be critical for full understanding of salt-tolerant mechanisms in alkaligrass.

In this study, we identified 25 *PutCrRLK1L* family genes in *P. tenuiflora* through genome-wide screening. We analyzed their phylogenetic relationships, chromosomal distribution, gene structures, conserved functional domains, and subcellular localization. Furthermore, we characterized *PutCrRLK1L* expression patterns under various salinity stress conditions. Phenotypic and physiological analyses of *PutFER1*-overexpressing lines demonstrated that *PutFER1* plays an essential role in root growth and ROS scavenging during salt stress adaptation.

## Materials and methods

### Identification and cloning of *PutCrRLK1L* genes in *P. tenuiflora*

Genome, transcripts, coding sequences (CDS), and peptide sequences data of *P. tenuiflora* were downloaded from the *P. tenuiflora* Ensembl database (http://www.xhhuanglab.cn/data/alkaligrass.html). Seventeen CrRLK1Ls protein sequences from *Arabidopsis thaliana* (including FER and its relatives) were downloaded from TAIR (https://www.arabidopsis.org/). We employed two complementary approaches to identify putative *PutCrRLK1L* genes in alkaligrass: (I) BLAST Search: 17 Arabidopsis CrRLK1L protein sequences were used as queries to search the alkaligrass protein database using the Basic Local Alignment Search Tool (BLAST). Putative *PutCrRLK1L* genes were screened based on sequence homology. (II) HMMER Scan: The Malectin-like domain (MLD, PF12819) and Protein kinase domain (Pkinase, PF07714) Hidden Markov Model (HMM) profiles were retrieved from the Pfam database (https://pfam.xfam.org). These profiles were used to scan the alkaligrass protein database using HMMER 3.0 software. Candidate sequences identified through both methods were subsequently validated using the Conserved Domain Database (CDD) tool (https://www.ncbi.nlm.nih.gov/cdd) from the National Center for Biotechnology Information (NCBI). Finally, each putative PutCrRLK1L protein sequence was manually verified using the Simple Modular Architecture Research Tool (SMART, http://smart.embl-heidelberg.de/) to confirm the presence of both the conserved malectin-like domain and protein kinase domains. The protein sequences of 25 PutCrRLK1Ls were displayed in **Supplementary Table 2**.

### Phylogenetic analysis of CrRLK1Ls from different plant species

Multiple sequence alignments were performed using Multiple Sequence Comparison by Log-Expectation (MUSCLE) v5.0 software with 904 CrRLK1L family members from 30 species. A phylogenetic tree was constructed from the CrRLK1L sequence alignment using FastTree v2.1 software. The resulting tree files were then submitted to iTOL (https://itol.embl.de/) for further modification.

### Collinearity analysis of *CrRLK1Ls* between alkaligrass and other plants

Collinearity analysis of the *CrRLK1Ls* between the alkaligrass and other plants was verified and visualized using One Stem MCScanX and Dual Systeny Plot for MCScanX in TBtools-II software, respectively.

### Analyses of gene structure and conserved motifs of *PutCrRLK1Ls*

The exon-intron structure of each *PutCrRLK1L* gene was examined using TBtools-II, based on its genomic sequence and CDS. Conserved motifs of 25 PutCrRLK1L proteins (with a maximum of 10 motifs) were identified using (Multiple Em for Motif Elicitation) MEME (https://meme-suite.org/meme/tools/meme) and visualized using TBtools-II.

### Identification of *cis*-regulatory elements in *PutCrRLK1Ls* promoters

Promoter sequences (2,000 bp upstream of the start codon) of all *PutCrRLK1Ls* were extracted from the *P. tenuiflora* Ensembl database (http://www.xhhuanglab.cn/data/alkaligrass.html). Putative *cis*-regulatory elements were identified using PlantCARE (http://bioinformatics.psb.ugent.be/webtools/plantcare/html/) and visualized using TBtools-II.

### RNA extraction and RT-qPCR

Total RNA was isolated from two-week-old seedlings using an RNAiso Plus reagent (TaKaRa, China) and was subsequently reverse transcribed using the TransScript^®^ One-Step gDNA Removal and cDNA Synthesis SuperMix Kit (TaKaRa, China). Quantitative PCR analysis was conducted with PerfectStart^®^ Green Quantitative Polymerase Chain Reaction SuperMix (TransGen Biotech, China) on a LC96 Real-Time System (Roche, Germany). RT-qPCR analyses were performed with three independent biological replicates, and the data were normalized using the 2^−ΔΔCt^ method (Livak and Schmittgen, 2001). The primer sequences are provided in **Supplementary Table 6**.

### CrRLK1Ls 3D protein structure prediction

The 3D protein structures of CrRLK1L proteins from different plant species were predicted using the AlphaFold database (https://alphafold.ebi.ac.uk/). Automatic modeling mode was selected within the AlphaFold interface and predicted protein structures were visualized using PyMOL software.

### Vector construction and plant transformation

The pCAMBIA1300-GFP plasmid was digested with *XbaI* and *SmaI*. The target *PutFER1* CDS fragment was amplified using alkaligrass cDNA as template and inserted into the digested pCAMBIA1300-GFP vector via homologous recombination at 37 °C for 30 minutes. The recombinant plasmid was transformed into *E. coli* DH5α. A sequence-verified positive clone was then transformed into *Agrobacterium tumefaciens* strain GV3101. GV3101 cells harboring the pCAMBIA1300-*PutFER1*-GFP plasmid were introduced into Arabidopsis wild-type seedlings via the floral dip method to generate *PutFER1*-overexpressing lines (Zhang et al., 2006). Transformed seedlings were selected on 1/2 MS agar medium containing 50 μg/mL hygromycin.

### Confocal imaging

Confocal microscopy was performed using a Leica STELLARIS 8 confocal laser scanning microscope. Green fluorescent protein (GFP) was excited at wavelengths of 488 nm and detected with a 500–550 nm spectral range.

### NBT staining assay

Accumulation of O_2_^•−^ in plant leaves was detected by nitroblue tetrazolium (NBT) staining as previously described (Zhao et al., 2021). Briefly, Arabidopsis seeds were germinated vertically on 1/2 MS agar plates for five days. Seedlings were then transferred to liquid 1/2 MS medium and incubated overnight with gentle shaking. After treatment with 150 mM NaCl for one hour, seedlings were stained with NBT solution (1 mg/mL NBT dissolved in distilled water) and incubated overnight at room temperature in the dark. Finally, stained seedlings were destained in ethanol overnight and visualized using a Leica EZ4 HD microscope.

### Determination of antioxidant enzyme activity

Antioxidant enzyme activities were determined using commercially available kits. In brief, total seedlings (0.1 g FW) were homogenized in the respective extraction buffer provided with the assay kit. The homogenate was centrifuged at 8,000 g for 10 min, and the supernatant was used as the enzyme extract for measurement. The activities of superoxide dismutase (SOD), peroxidase (POD), catalase (CAT), and ascorbate peroxidase (APX) in wild-type and two *PutFER1*-overexpressing Arabidopsis lines were determined using Solarbio Assay Kits (Kit Codes: BC0170, BC0090, BC0200, and BC0220) following the manufacturer’s instructions (Solarbio, China).

### Quantification and statistical analysis

All graphs were generated using GraphPad Prism v8.0 for Windows. Statistical analyses were performed using Student’s *t*-test (two-sided) for two groups or one-way analysis of variance (ANOVA) followed by Duncan analysis for multiple groups in IBM SPSS Statistics 25.0 software (https://www.ibm.com/products/spss-statistics). A *P* value less than 0.05 was considered significant.

## Results

### CrRLK1L gene family expanded and FER is conserved during plant evolution

**Figure 1:**
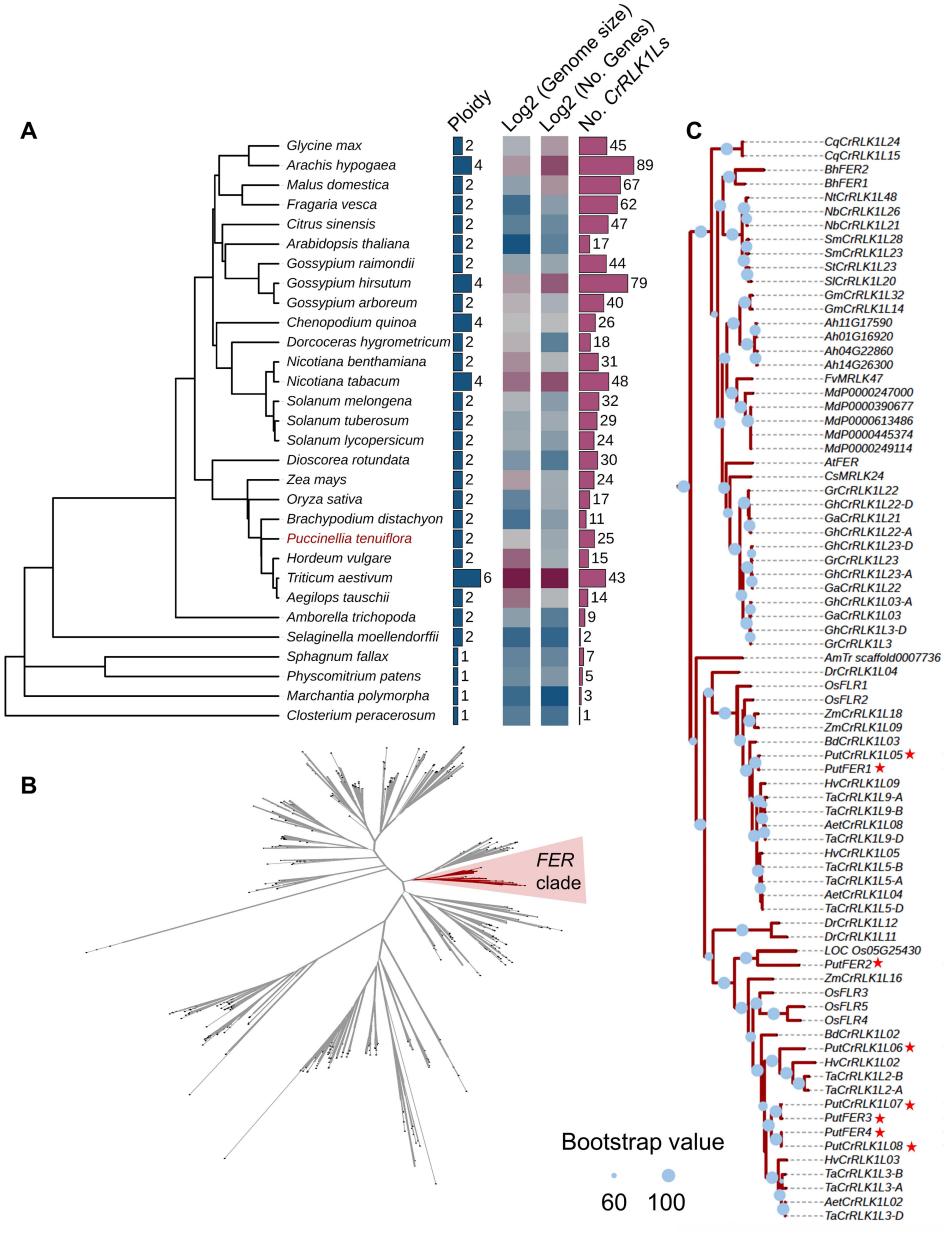
Phylogenetic analysis of *Catharanthus roseus* RLK1-Like (*CrRLK1L*) and *FERONIA* (*FER*) in different species. **(A)** Analysis of phylogenetic tree, ploidy, genome size, and gene and *CrRLK1L* numbers in diverse species. **(B)** CrRLK1L phylogenetic tree analysis in diverse species. The phylogenetic tree was constructed using the amino acid sequences of the CrRLK1Ls with FastTree, which indicates the phylogenetic relationships among 904 CrRLK1Ls from diverse species. The red colored ranges indicate the FER clade. Branches with a bootstrap value > 0.6 are indicated by thick lines. **(C)** FER phylogenetic tree. The phylogenetic tree was built based on the amino acid sequences from the FER clade in **(B)**. The red star represents the alkigrass *PutCrRLK1L* members. Blue circles represent bootstrap values. They are displayed on branches with Bootstrap > 60, where a larger circle indicates higher support.

We comprehensively analyzed the genomes and 904 *CrRLK1L* genes across 30 phylogenetically diverse plant species with sequenced genomes (**Figure 1A**, **Supplementary Table 1**). The genome analysis indicated their evolutionary relationships among algae, moss, ferns, early angiosperm *Amborella*, monocotyledon, and dicotyledon (**Figure 1A**). Most studies of *CrRLK1L* gene family focused on Solanaceae (five plant species) and Poaceae (seven plant species) (**Figure 1A**, **Supplementary Table 1**). Among these species, unicellular charophycean alga (*Closterium peracerosum-strigosum-littorale* complex), liverwort (*Marchantia polymorpha*), moss (*Physcomitrella patens*), and bog moss (*Sphagnum fallax*) are haploid, tobacco (*N. tabacum*), cotton (*Gossypium hirsutum*), quinoa, and peanut (*Arachis hypogaea*) are tetraploid, while the remaining 21 plant species are diploid. Overall, the number of *CrRLK1Ls* in tetraploid and hexaploid plants was larger than those in most diploid and haploid plants (**Figure 1A**). The number of *CrRLK1L* copies ranges from one in unicellular charophycean alga to 89 in peanut (**Figure 1A**, **Supplementary Table 1**). Importantly, the study of *CrRLK1Ls* across eight monocotyledonous and 16 dicotyledonous plant species revealed that wheat (*T. aestivum*, *TaCrRLK1Ls*) from the Poaceae family and peanut (*A. hypogaea*, *AhCrRLK1Ls*) from the Fabaceae family were the most extensively studied, with 43 and 89 *CrRLK1Ls* investigated, respectively (**Figure 1A**). These findings indicate that *CrRLK1L* genes are widely distributed among different plant species and that this gene family has undergone lineage-specific expansion during the evolution of plant species.

Given that FER is the most widely studied and best characterized CrRLK1L family protein, we explored its evolution within the CrRLK1L gene family by constructing a phylogenetic tree using the full-length sequences of all 904 CrRLK1L proteins from 30 published plant species (**Figures 1B, C**, **Supplementary Table 1**). The phylogenetic tree reveals that *FER* genes from vast majority plant species are classified into a single clade, indicating that *FER* has maintained structural homology throughout its evolutionary history (**Figure 1B**). Notably, we identified 25 *CrRLK1L* members in *P. tenuiflora*, including eight FER orthologs of *A. thaliana* (**Supplementary Table 2**). Among them, we cloned four *PutFER* genes (*PutFER1* ~ *4*) and verified their sequences by DNA sequencing (**Supplementary Table 2**). Additionally, the copy number of *FER* genes varies across species, ranging from one in the *Amborella trichopoda* genome to eight in the *P. tenuiflora* genome and nine in the *T. aestivum* genome, indicating that the *FER* gene family has expanded during species evolution, and that whole-genome duplication events triggered the expansion (**Figure 1C**; **Supplementary Table 1**). Eight *FER* genes from four species (*C. psl.* complex, *M. polymorpha*, *S. fallax*, and *P. patens*) out of the 30 species do not fall within this clade (**Figure 1C**). These four species represent basal lineages that diverged prior to *Am. trichopoda*, one of the earliest diverging angiosperms. This suggests that the *FER* gene family likely originated after the divergence of angiosperms and subsequently underwent lineage-specific expansion. In conclusion, our results indicate that FER underwent expansion during plant evolution, while it was conserved during this process.

### Chromosomal distribution and collinearity analysis of *PutCrRLK1Ls*

**Figure 2:**
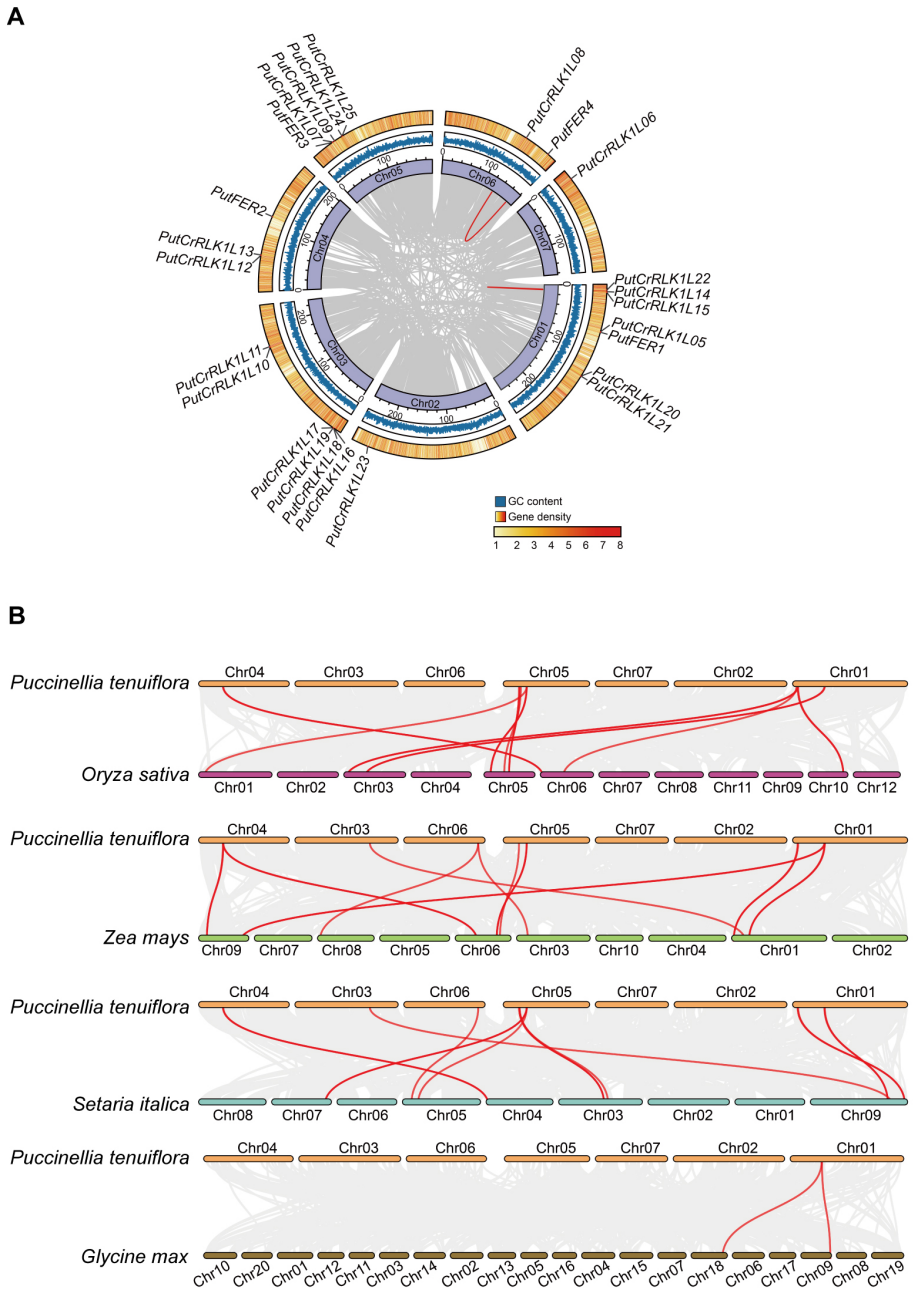
Distribution of *PutCrRLK1Ls* on chromosomes and collinearity analysis of *CrRLK1Ls* between *Puccinellia tenuiflora* and other plant species. **(A)** Distribution of *PutCrRLK1Ls* in chromosomes and duplication events analysis of *PutCrRLK1Ls*. Gray lines in the background indicate the collinear blocks within *P. tenuiflora*, while the red lines highlight the *PutCrRLK1L* gene pairs that are syntenic. **(B)** Collinearity analysis of *PutCrRLK1Ls* among alkaligrass (*P. tenuiflora*), rice (*Oryza sativa*), maize (*Zea mays*), foxtail millet (*Setaria italica*), and soybean (*Glycine max*). Grey lines in the background indicate the collinear blocks within *P. tenuiflora* and other plant genomes, while the red lines highlight the syntenic *CrRLK1L* gene pairs.

Chromosome distribution analysis revealed that 25 *PutCrRLK1Ls* exhibited an uneven and non-random distribution across the seven chromosomes in alkaligrass (**Figure 2A**). Chromosome 1 harbored seven *PutCrRLK1Ls*, chromosome 3 contained six, chromosome 5 had five, chromosome 4 had three, chromosome 6 had two, and chromosomes 2 and 7 each contained one *PutCrRLK1L* (**Figure 2A**). Among them, four *PutFERs* were evenly distributed on chromosomes 1, 5, and 6. These results indicate that *PutCrRLK1Ls* may have undergone functional divergence during evolution and developed independent gene expression regulation, thereby enhancing alkaligrass adaptation to the environments (**Figure 2A**).

To gain insight into the expansion pattern of the PutCrRLK1L gene family, we analyzed the duplication events using MCScanX (**Figure 2A**). This analysis identified two highly homologous gene pairs (*PutCrRLK1L08*/*PutFER4* and *PutCrRLK1L14*/*PutCrRLK1L15*) that exhibited high levels of homozygosity, with sequence identity of 97.35% and 99.53%, respectively. Furthermore, the non-synonymous substitution rate (Ka) over the synonymous substitution rate (Ks) (Ka/Ks values) in these gene pairs were less than one (**Supplementary Table 3**). This demonstrates that CrRLK1L gene family in alkaligrass has undergone purifying selection during its expansion process.

To further understand the evolutionary mechanisms of *PutCrRLK1Ls*, a collinearity analysis was performed to compare the homologous *CrRLK1L* genes among alkaligrass, rice (*Oryza sativa*), maize, foxtail millet (*Setaria italica*), and soybean (**Figure 2B**). The results revealed that alkaligrass *PutCrRLK1Ls* had 15, 16, 14, and two homologous gene pairs in rice, maize, foxtail millet, and soybean, respectively (**Figure 2B**, **Supplementary Table 4**). On the other hand, maize, rice, foxtail millet, and soybean *CrRLK1Ls* have ten, nine, nine, and two genes exhibiting synteny with *PutCrRLK1Ls*, respectively (**Figure 2B**, **Supplementary Table 4**). These findings indicate that the *CrRLK1L* genes in alkaligrass share stronger phylogenetic relationships with orthologs from monocotyledons such as rice and maize than with those from dicotyledons such as soybean.

### Gene structure and protein function domain analysis of *PutCrRLK1Ls*

**Figure 3:**
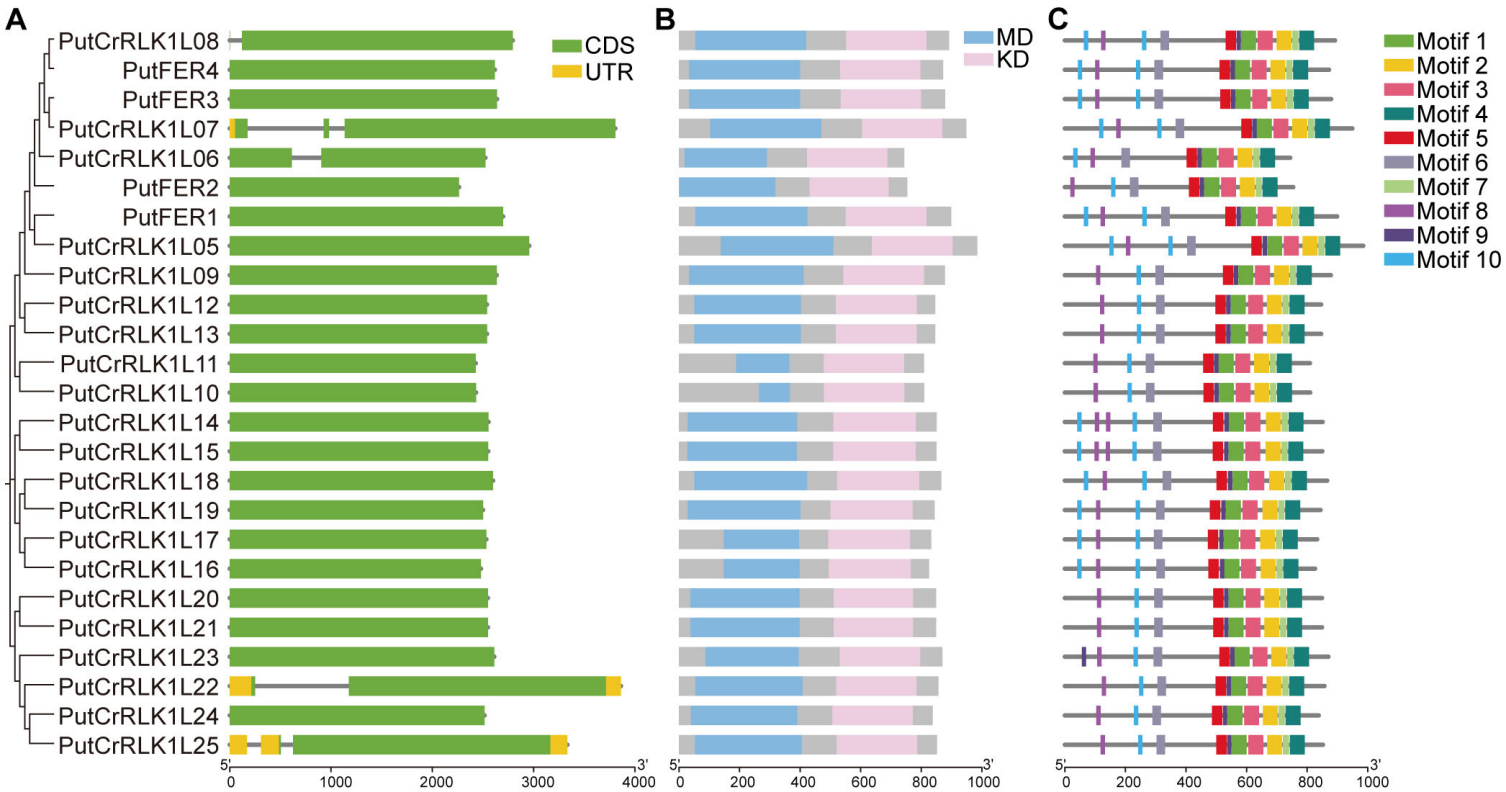
Gene structure, protein domain, and conserved motifs of PutCrRLK1Ls. **(A)** The structure of *PutCrRLK1Ls* includes exons, introns, and untranslated regions (UTRs). Yellow boxes indicate UTR, gray lines indicate introns, and green boxes indicate exons. **(B)** Protein domain of *PutCrRLK1Ls*. Blue boxes indicate malectin domain (MD) and pink boxes indicate kinase domain (KD). **(C)** Conserved motifs in *PutCrRLK1Ls*. Different colors represent different motifs.

To further elucidate the evolutionary relationships of the PutCrRLK1L gene family, we conducted a comprehensive analysis of gene structures, conserved protein domain and motifs. Our results reveal that only three genes (*PutCrRLK1L07*, *PutCrRLK1L22* and *PutCrRLK1L25*) possess both a 5’ UTR and a 3’ UTR, whereas the remaining *PutCrRLK1Ls* lack UTRs (**Figure 3A**). Additionally, five genes (*PutCrRLK1L06*, *PutCrRLK1L07*, *PutCrRLK1L08*, *PutCrRLK1L22*, and *PutCrRLK1L25*) have introns, whereas the others do not (**Figure 3A**). The conserved and simplified gene architecture of *PutCrRLK1Ls* members suggests that they may enhance gene expression efficiency by reducing alternative splicing processes, thereby enabling a rapid response to fluctuating environments.

Protein domain analysis indicates that PutCrRLK1L family members possess conserved extracellular malectin domains and intracellular kinase domain (**Figure 3B**). These results provide strong evidence for the functional conservation of PutCrRLK1Ls throughout evolution. Furthermore, we predicted the distribution of conserved motifs in PutCrRLK1L proteins and identified ten conserved motifs (**Figure 3C**, **Supplementary Figure 1**). Notably, motifs one to ten are universally present in the majority of PutCrRLK1L family members, suggesting they play a crucial role in maintaining structural or functional integrity (**Figure 3C**). However, an interesting observation is that the number of motifs eight and ten, which are extracellular MLDs, varies (two to four copies) among PutCrRLK1L family members, suggesting potential functional divergence in signal perception and interaction with other molecules (**Figure 3C**). Motifs one, two, three, four, five, seven, and nine, which are located in the intracellular domain, are present in all PutCrRLK1L members (**Figure 3C**). Furthermore, the number and variety of intracellular motifs exceed that of extracellular motifs (**Figure 3C**). Taken together, our findings suggest that these proteins may play a significant role in maintaining intracellular signaling homeostasis during development and in response to environmental stress in alkaligrass.

### Analysis of *cis*-acting elements in PutCrRLK1Ls promoters

**Figure 4:**
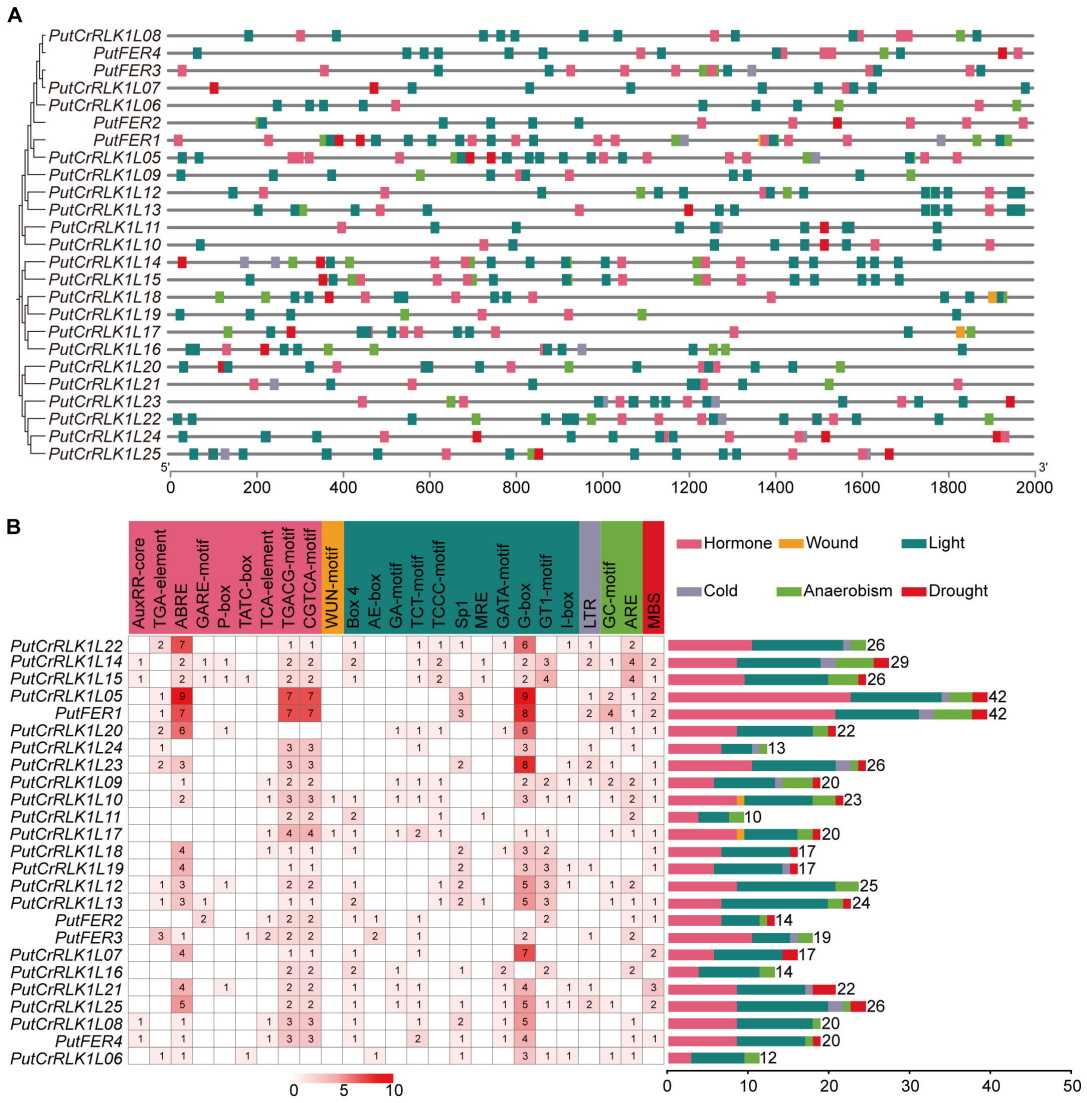
Analysis of *cis*-acting elements in the promoter region of the *PutCrRLK1Ls*. **(A)** The *cis*-acting elements distribution in *PutCrRLK1Ls* promoter regions. **(B)** The names and numbers of *cis*-acting elements in *PutCrRLK1Ls* promoters. The heatmap in grid and the color columns indicated the numbers of *cis*-acting elements. ABRE, ABA responsive element; ARE, Anaerobic-responsive element; GARE, Gibberellin-responsive element; LTR, Low temperature-responsive element; MBS, MYB-binding site; MRE, Metal response element.

To investigate the potential functions of the 25 *PutCrRLK1L* genes, *cis*-acting element prediction was performed on their promoter regions and a schematic map showing the prevalent distribution of stress- and hormone-responsive *cis*-acting elements was created (**Figure 4A**; **Supplementary Table 5**). Overall, the 25 *PutCrRLK1L* genes in alkaligrass show significant enrichment of phytohormones (nine elements) and light (11 elements) (**Figure 4B**). Certain elements are responsive to phytohormones (e.g., ABRE, TGACG motif and CGTCA motif), light (e.g., G-box, Sp1 and GT1 motif), anaerobism (e.g., GC motif and ARE) and drought (e.g., MBS). Some of these elements are conserved among the 25 *PutCrRLK1Ls* (**Figure 4B**). These findings suggest that *PutCrRLK1Ls* play a vital role in plants response to drought, light, anaerobism, and hormonal signals. Notably, a total of 234 light-responsive elements were identified. Additionally, 227 hormone-responsive elements were detected, including 70 abscisic acid (ABA) elements (also known as ABREs), 116 jasmonate elements (specifically TGACG and CGTCA motifs), nine salicylic acid elements (identified as the TCA element), 13 gibberellin elements (specifically GARE, P-box, and TATC-box elements), and 19 auxin elements (specifically TGA elements and AuxRR-core elements) (**Figure 4B**). The conserved elements found in the promoter region suggest that *PutCrRLK1Ls* play a crucial role in responding to abiotic stress and phytohormone treatment.

### Transcriptional analysis of *PutFERs* in response to salt stress

**Figure 5:**
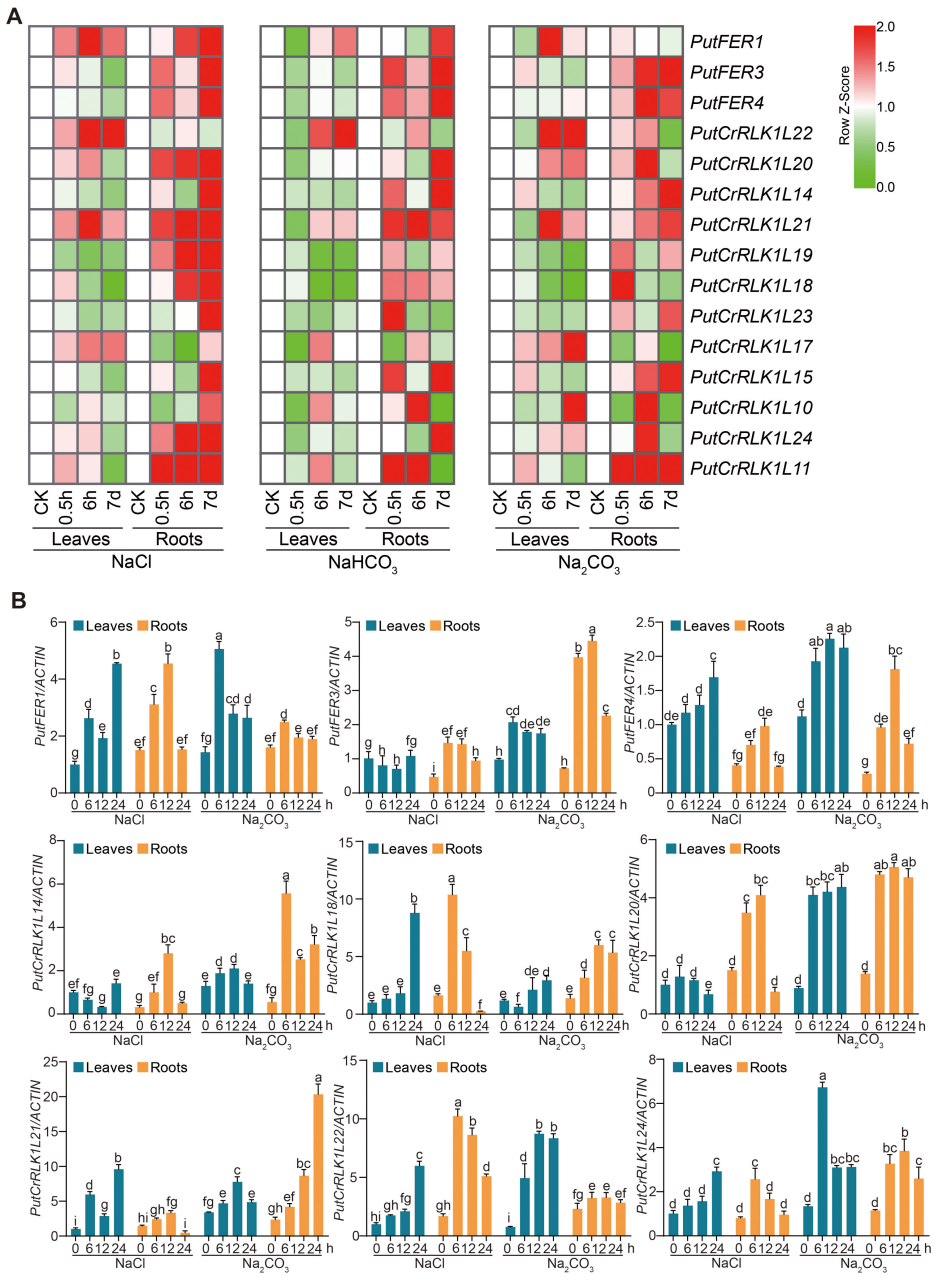
Salt stress induces *PutFER1* in alkaligrass roots and leaves. **(A)** The heat map of *PutCrRLK1Ls* expression in root and leaf samples treated with NaCl, NaHCO_3_, and Na_2_CO_3_ for 0.5 h, 6 h, and 7 d, based on gene expression profiles. Red and green colors indicated up- and down-regulated genes, respectively. The color scale in the heatmap indicates the relative expression level of each gene across leaves and roots, based on the row Z-score of FPKM values. **(B)** Expression levels of *PutCrRLK1Ls* in leaves and roots using RT-qPCR analysis under salt and alkali stress conditions. The RT-qPCR results were calculated using the 2^−ΔΔCt^ method, with *PutACTIN* serving as reference genes for normalization. The expression levels of *PutCrRLK1Ls* were analyzed in 14-day-old alkaligrass seedlings treated with 200 mM NaCl or 100 mM NaHCO_3_ (pH 9.0) for the indicated durations. Different letters indicate statistically significant differences (*P* < 0.01, one-way ANOVA).

Given the pivotal role of Arabidopsis FER in regulating plant salt stress responses (Feng et al., 2018), we were interested in elucidating whether *PutFERs* in alkaligrass are also required for salt tolerance. We have performed a series of deep transcriptome sequencing treated with NaCl, Na_2_CO_3_, and NaHCO_3_ using the Illumina sequencing platform (Zhang et al., 2020). Based on the transcriptome sequencing data, we analyzed the expression profiles of *PuCrRLK1L* family members in roots and leaves under different salt stress conditions (**Figure 5A**). Overall, most PutCrRLK1L family genes were significantly more highly expressed in roots than in leaves under all salt stress conditions (**Figure 5A**).

In roots, *PutFER3* and *PutCrRLK1L11* were significantly induced to express under all salt stress conditions, whereas *PutFER4*, *PutCrRLK1L18*, and *PutCrRLK1L21* were increased under NaCl and NaHCO_3_ treatments. Interestingly, *PutCrRLK1L17* and *PutCrRLK1L22* were initially reduced and subsequently induced under NaCl and NaHCO_3_ treatments, whereas they induced under Na_2_CO_3_ treatment (**Figure 5A**).

In leaves, the expression of four genes (*PutFER1*, *PutCrRLK1L17*, *PutCrRLK1L21*, and *PutCrRLK1L22*) were induced under NaCl conditions, while that of five genes (*PutFER4*, *PutCrRLK1L14*, *PutCrRLK1L15*, *PutCrRLK1L19*, and *PutCrRLK1L23*) were reduced (**Figure 5A**). Under Na_2_CO_3_ conditions, only *PutCrRLK1L24* was induced, while three genes (*PutCrRLK1L18*, *PutCrRLK1L19*, and *PutCrRLK1L23*) were reduced. Interestingly, under NaHCO_3_ conditions, all screened *PutCrRLK1L* genes were reduced at 0.5 h (**Figure 5A**). Additionally, *PutFER1* and *PutCrRLK1L21* were significantly induced in both leaves and roots under NaCl conditions.

We subsequently selected nine *PutCrRLK1L* genes that showed significant differential expression under saline-alkali stress and analyzed their transcript levels using RT-qPCR. The results revealed that the majority of these genes were significantly induced by saline-alkali stress in both leaves and roots (**Figure 5B**; **Supplementary Figures 2A, B**). In addition, the expression of *PutFER3* and *PutCrRLK1L14* in leaves was significantly downregulated, which is consistent with the trend observed in the transcriptome data.

### Protein structure and subcellular localization of PutFER1

**Figure 6:**
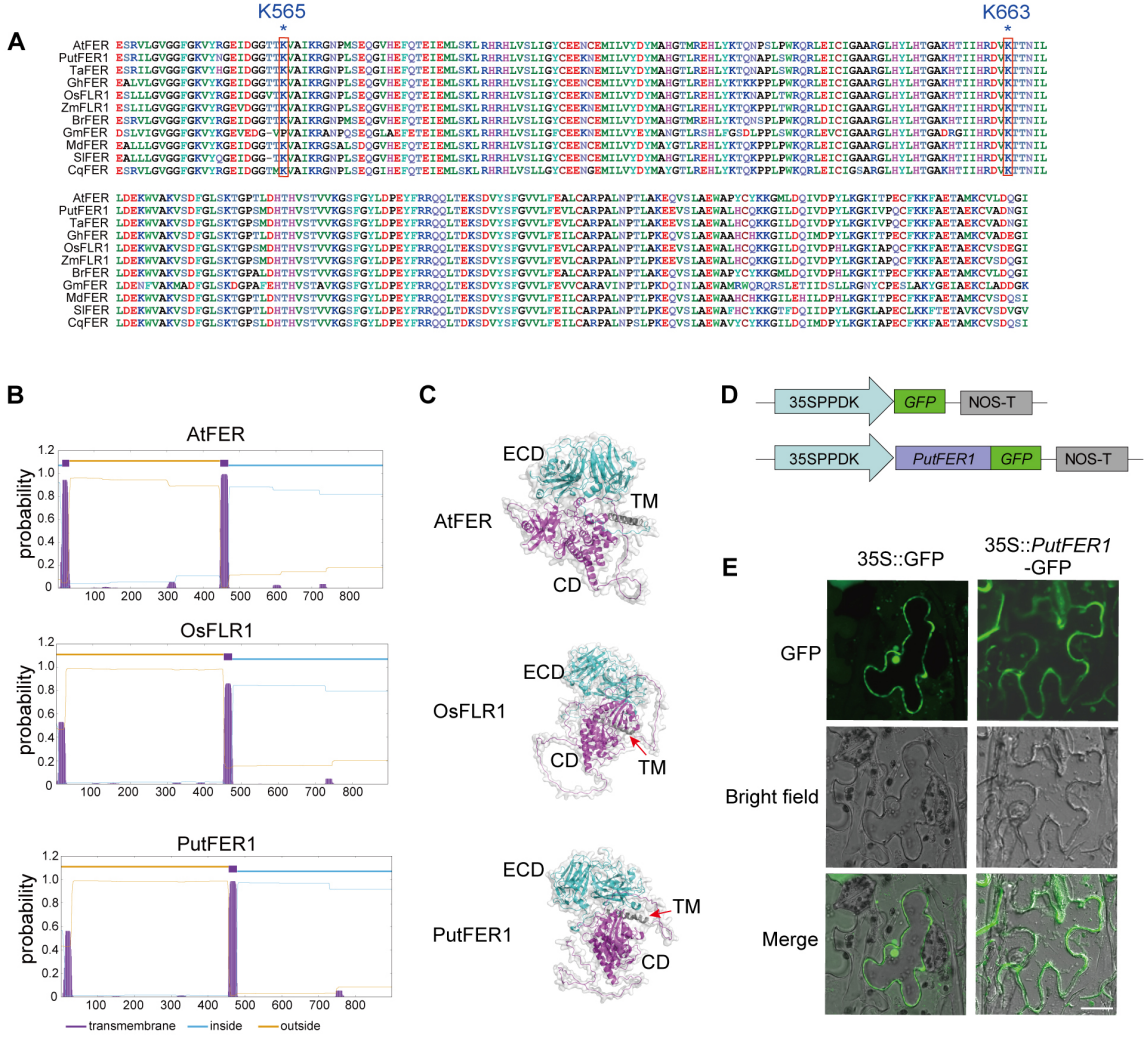
Protein structure and subcellular localization of PutFER1. **(A)** Amino acid sequence alignment of the kinase domain of FER proteins from 11 plant species. The conserved active sites of Arabidopsis K565 and K663 in 11 plant species are marked with red boxes. Sequence alignment was performed using ClustalW, and results were visualized using Bioedit. **(B)** Prediction of transmembrane domains in PutFER1 protein. Purple, lightblue, and yellow represent transmembrane domain (TM), intracellular regions, and extracellular regions, respectively. **(C)** Predicted 3D protein structure of PutFER1. Light blue, gray, and purple represent the extracellular domain (ECD), TM, and cytoplasmic domain (CD), respectively. The structure was predicted using AlphaFold and visualized using PyMOL software. **(D)** Schematic diagrams of control construct (35S::GFP) and recombinant construct (35S::*PutFER1-*GFP). 35SPPDK, hybrid promoter consisting of the cauliflower mosaic virus (CMV) 35S enhancer fused to the maize C4PPDK basal promoter. NOS-T, Nopaline synthase terminator. **(E)** Transient expression of GFP and *PutFER1*-GFP in tobacco (*Nicotiana benthamiana*) leaf epidermal cells. Scale bar, 20 µm.

To further investigate the function of PutFER1, we conducted a conservation analysis of kinase active sites within the FER kinase domain, specifically residues K565 and K663 (based on the Arabidopsis sequence), using published data (Minkoff et al., 2017). The sequence alignment among FERs from 11 plant species revealed that the kinase domain and the active sites are highly conserved (**Figure 6A**). This implied that PutFER1 could play a conserved role in maintaining the intracellular downstream signaling cascade (**Figure 6A**).

As a receptor-like kinase, Arabidopsis FER possesses a typical single-pass transmembrane domain (Li et al., 2015). We predicted and compared the hydrophobic transmembrane region between PutFER1 and other 10 species (**Figure 6B**; **Supplementary Figure 3**). As shown in **Figure 6B**, PutFER1 has a typical single-pass transmembrane domain, similar to those found in rice and Arabidopsis (**Figure 6B**). The 3D protein structures of PutFER1 and the other ten plant species indicated that the extracellular domain of PutFER1 contains a high proportion of beta-sheets, while the intracellular domain is rich in alpha-helices (**Figure 6C**; **Supplementary Figure 4**). These results suggest that PutFER1 may play a key role in enhancing the efficiency of extracellular ligand recognition and intracellular signal transduction.

To explore the subcellular localization of PutFER1, full-length coding DNA sequence (CDS) of *PutFER1* was cloned into the binary vector pCAMBIA1300-GFP and resulting in translational fusion with green fluorescent protein (GFP) (**Figure 6D**). GFP signal was observed at the plasma membrane using confocal fluorescence imaging in the tobacco (*Nicotiana benthamiana*) system, suggesting that PutFER1 is located on the plasma membrane (**Figure 6E**).

### PutFER1 is essential for plant salt tolerance and ROS scavenging

**Figure 7:**
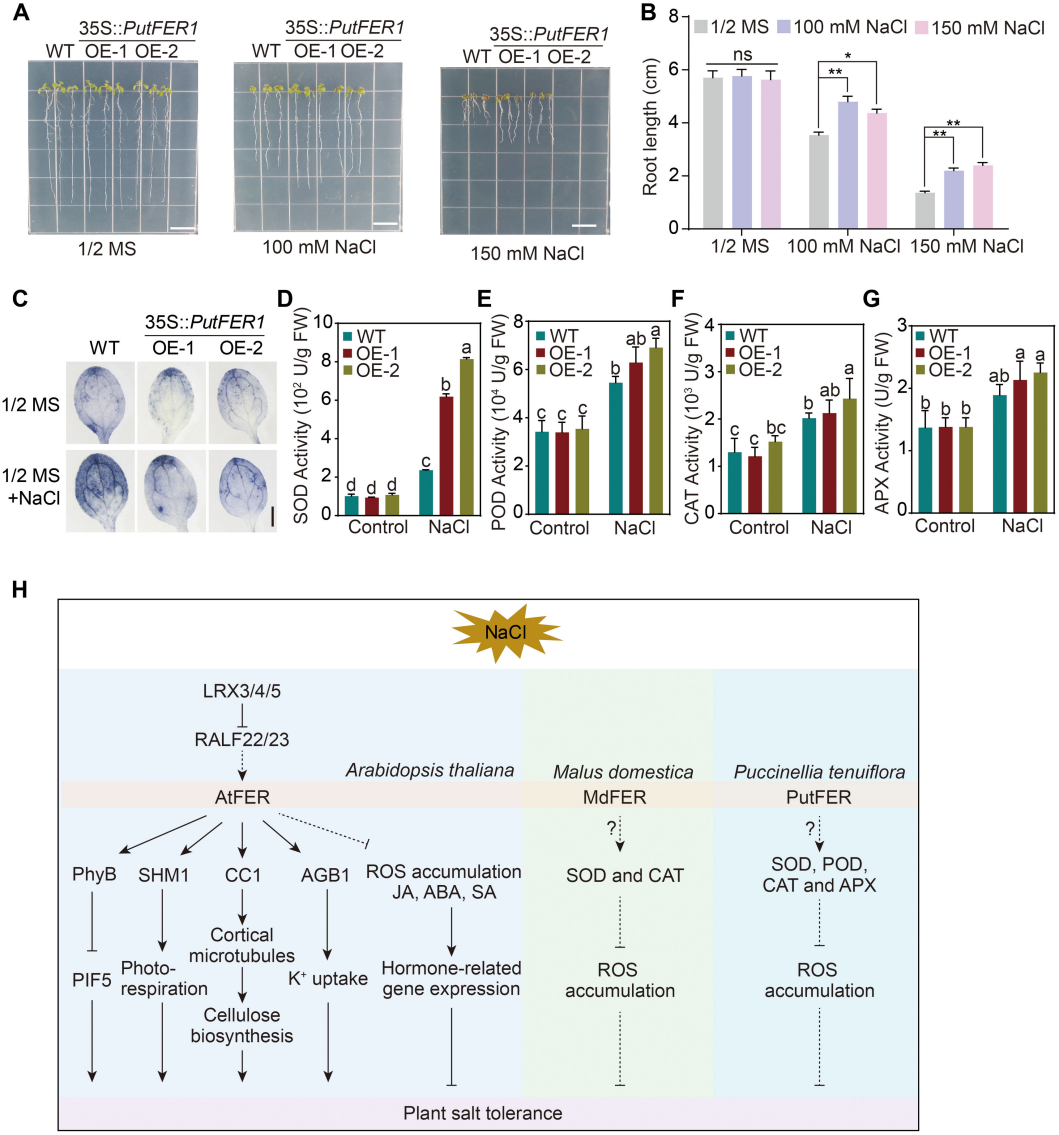
The salt-responsive phenotypes of wild-type, *PutFER1*-overexpressing Arabidopsis plants. **(A)** The salt-responsive phenotypes of wild-type and two lines of *PutFER1*-overexpressing Arabidopsis. Seedlings were grown on 1/2 MS medium for 3 d, and then transferred to 1/2 MS medium with or without NaCl for 5 d. Scale bars, 1 cm. **(B)** Quantification of root length shown in **(A)**. Data represent means ± S.D. (n=15 in each biological replicates). ** *P* < 0.01 (two-tailed Student’s *t*-test). **(C)** The accumulation of O_2_^•−^ in the leaves from five-day-old Arabidopsis seedlings. The O_2_^•−^ levels from wild-type and *PutFER1*-overexpressing Arabidopsis plants under 0 mM and 150 mM NaCl treatment were detected using NBT staining method. Scale bars, 0.2 cm. **(D–G)** Activities of superoxide dismutase (SOD), peroxidase (POD), catalase (CAT), and ascorbate peroxidase (APX) wild-type and two lines of *PutFER1*-overexpressing Arabidopsis under control and NaCl conditions. Different letters indicate statistically significant differences (*P* < 0.01, one-way ANOVA). **(H)** A proposed model illustrating FER-mediated intracellular signaling pathways in response to salt stress in *Arabidopsis thaliana*, *Puccinellia tenuiflora*, and *Malus domestica*. In *Arabidopsis thaliana*, salt stress inhibits AtFER kinase activity, leading to the accumulation of phyB in the nucleus, which subsequently suppresses PIF5-mediated plant growth. AtFER enhances photorespiratory flux by phosphorylating SHM1, thereby promoting growth under salt stress. Additionally, AtFER promotes cortical microtubule polymerization and subsequent cellulose synthesis by phosphorylating CC1, further supporting plant growth during salt stress. AtFER, together with AGB1, coordinately regulates K^+^ uptake under salt stress. The LRX3/4/5-RALF22/23-FER module enhances salt stress tolerance by suppressing intracellular ROS levels and modulating hormone signaling (JA, ABA, and SA). In *Puccinellia tenuiflora*, PutFER1 contributes to salt stress adaptation by increasing the activity of antioxidant enzymes (SOD, POD, CAT, and APX), which scavenge excess ROS. Similarly, in *Malus domestica*, MdFER improves salt stress tolerance by enhancing the activities of SOD and CAT to mitigate ROS accumulation. ABA, Abscisic Acid; AGB1, Arabidopsis G protein β subunit; APX, Ascorbate peroxidase; CAT, Catalase; CC1, COMPANION OF CELLULOSE SYNTHASE 1; FER, FERONIA; JA, Jasmonic Acid; LRX3/4/5, LEUCINE-RICH REPEAT EXTENSINS 3/4/5; PhyB, PHYTOCHROME B; PIF5, PHYTOCHROME INTERACTING FACTOR 5; POD, Peroxidase; RALF22/23, RAPID ALKALINIZATION FACTOR 22/23; ROS, Reactive oxygen species; SA, Salicylic Acid; SHM1, Serine Hydroxymethyltransferase 1; SOD, Superoxide dismutase.

In Arabidopsis, FER was shown to be involved in salt tolerance (Zhao et al., 2018), so we were interested in elucidating whether PutFER1 in alkaligrass is also required for salt tolerance. We then generated a *PutFER1* construct driven by 35S promoter and transformed it into wild-type Arabidopsis plants. RT-qPCR analysis revealed that the other two transgenic lines exhibited transcript levels >3-fold higher than the line with the lowest expression (**Supplementary Figure 5**), confirming successful transgene expression. Phenotype analysis revealed that overexpressing *PutFER1* in Arabidopsis results in an enhanced root length under salinity conditions (**Figures 7A, B**), suggesting that *PutFER1* in alkaligrass participates in regulating salt tolerance. Additionally, the Arabidopsis *fer-4* mutant has been reported to exhibit ROS overaccumulation, which drives salt-induced cell death (Zhao et al., 2021). We assessed ROS homeostasis in the wild-type and two *PutFER1*-overexpressing Arabidopsis lines. Lower levels of O_2_^•−^ were observed in the *PutFER1*-overexpressing lines than in the wild type (**Figure 7C**), indicating that *PutFER1* regulates intracellular ROS homeostasis during salt stress responses. To investigate the physiological impact of *PutFER1*-overexpression, we assessed the activities of four key antioxidant enzymes including SOD, POD, CAT, and APX in wild-type and *PutFER1*-overexpressing lines under both normal and salt-stress conditions. The results revealed that salt stress induced a marked increase in the activities of all four enzymes in the two overexpression lines compared to the WT (**Figures 7D–G**). These findings suggest that *PutFER1* may enhance salt stress tolerance by boosting the activity of various antioxidant enzymes to scavenge excess ROS.

## Discussion

### Genomic expansion and functional adaptation of the CrRLK1L family in *P. tenuiflora*

As important members of the plant RLK family, CrRLK1Ls have been identified in 29 species, including angiosperms (e.g., Arabidopsis, rice, and apple), gymnosperms (e.g., *Picea abies*), and early-diverging lineages (e.g., *C. psl.* complex, *M.polymorpha*, and *P. patens*) (Hirano et al., 2015; Bowman et al., 2017; Westermann et al., 2019; Zhu et al., 2021; Mecchia et al., 2022; Ma et al., 2023b). In this study, we comprehensively analyzed the structural characteristics, expression patterns, and biological functions of *CrRLK1Ls* in *P. tenuiflora*. Similar with some plant species, such as apple (Zuo et al., 2018) with 67 genes, tobacco (Li et al., 2022) with 48 genes, cotton (Niu et al., 2016) with 79 genes, and soybean (Jiang et al., 2024b) with 45 genes, the *PutCrRLK1L* family with 25 members has also expanded significantly. This expansion likely reflects genomic adaptations to the extreme saline-alkaline habitats of *P. tenuiflora*.

The presence or absence of introns, as well as their abundance, can reflect the evolution of plant genomes and the functional divergence of genes (Liu et al., 2021b). RNA-seq analyses of Arabidopsis and rice revealed that intronless or intron-poor genes, including the AP2, EF-hand, bZIP, FAD-binding and C2 gene families, play a greater role in responding to drought and salt stress than intron-rich genes in the same families (Liu et al., 2021b). Importantly, intronless genes in the S-domain RLK gene family are more likely to be involved in epigenetic processes and plant development (Liu et al., 2021b). We found alkaligrass *PutCrRLK1Ls* almost universally lack introns and UTRs (**Figure 3A**), which obviously contrasts with many other *CrRLK1L* families of maize, tomato (*Solanum lycopersicum*), yam (*Dioscorea rotundata*), and citrus (*Citrus sinensis*) (Luo et al., 2020; Ma et al., 2023b; Wang et al., 2024a; Qiao et al., 2025). For instance, approximately half of the maize *ZmCrRLK1L* family members have about 1 ~ 7 introns (Wang et al., 2024a). This pattern may imply an adaptive feature in *P. tenuiflora*, potentially enabling faster transcriptional activation and/or more efficient translation in response to rapidly changing environmental stresses such as salinity.

In addition, similar to those in maize (Wang et al., 2024a), soybean (Jiang et al., 2024b), and potato (Bao et al., 2025), the PutCrRLK1L family members exhibit a comparable pattern of variation in the number of MLD motifs (2 ~ 4). This suggests that PutCrRLK1Ls recognize signals differently in response to various environmental processes.

### Comparative *cis*-regulatory elements of *PutCrRLK1Ls*

Promoter ***cis***-elements play a crucial role in regulating gene expression patterns, stress responses, and biological functions (Marand et al., 2023). The promoters of *CrRLK1L* family members in multiple plant species (e.g., wheat, maize, and tomato) are enriched with various *cis*-acting elements responsive to hormones and abiotic stress (Ma et al., 2023b; Gawande and Sankaranarayanan, 2024; Wang et al., 2024a). For instance, 24 soybean *GmCrRLK1L* genes possessed the ABA-responsive element ABRE, while 12 and 11 *GmCrRLK1L* genes contained the drought-responsive element MBS and the low temperature-responsive element LTR, respectively (Wang et al., 2021). In addition, 29 potato *StCrRLK1L* genes contain at least four *cis*-elements related to light response, while 10 *StCrRLK1L* genes contain *cis*-elements related to phytohormones (Bao et al., 2025). In our studies, 25 *PutCrRLK1L* genes from alkaligrass exhibit significant enrichment of *cis*-elements related to phytohormones (nine elements) and light response (11 elements) (**Figure 4B**). In addition, at least 20 *PutCrRLK1Ls* contain both ABRE and light-responsive G-box elements, which is consistent with soybean *GmCrRLK1Ls* and potato *StCrRLK1Ls* (Jiang et al., 2024b; Bao et al., 2025). This indicates that *PutCrRLK1Ls* could be involved in hormone and light signaling pathways. Arabidopsis FER confers resistance to photooxidative stress and protects photosystems under moderate light conditions (Shin et al., 2021). Meanwhile, FER fine-tunes the balance between growth and stress by regulating the signaling of hormones such as ABA, JA, and SA (Zhao et al., 2021). Furthermore, the key transcription factor SlBZR1, which is involved in BR signaling, enhances thermotolerance in tomato by directly binding to the E-box (CANNTG) in the *SlFERs* promoter (Yin et al., 2018). However, no BR relevant regulatory elements were identified in the promoter region of the *PutFERs* in alkaligrass. Therefore, screening for transcription factors that directly bind to the *cis*-elements in the FER promoter could deepen our understanding of the FER-mediated signaling pathway.

### Functional significance of PutFER1 in salt stress adaptation

As a central member of the CrRLK1L family, FER is widely recognized for its functional versatility in plants, regulating processes span growth, reproduction, immunity, and responses to abiotic stresses such as salinity, drought, and heat (Yin et al., 2018; Huang et al., 2020b, 2023; Jing et al., 2023; Cheung, 2024; Jiang et al., 2024a). In glycophytes like Arabidopsis, FER acts as a polymodal integrator, coordinating salt tolerance through multiple interconnected pathways. FER perceives cell wall integrity via pectin binding, activates Ca²^+^ and MAPK signaling (Feng et al., 2018; Gigli-Bisceglia et al., 2022), modulates stomatal dynamics through G-protein interactions (Yu and Assmann, 2018), and fine-tunes hormonal and ROS homeostasis via leucine-rich repeat extensins (LRXs)-RALF modules (Zhao et al., 2021). Furthermore, FER regulates metabolic adaptation and growth under stress by targeting SHM1 and phyB-PIF signaling (Liu et al., 2023; Jiang et al., 2024a), thereby mediating a trade-off between stress adaptation and growth suppression. In halophytes such as quinoa (*Chenopodium quinoa*), FER homologs are rapidly induced under salt stress, indicating a conserved role in stress perception. All these findings suggest that the core FER-dependent pathways appear evolutionarily conserved, while the species-specific functional adaptations have emerged, such as regulating ABA sensitivity in apple (*Malus domestica*), ion homeostasis in quinoa, and ROS production in *Brassica rapa* (Zhang et al., 2021a; Bazihizina et al., 2022; Xie et al., 2022). This underscores FER’s versatile role in bridging salt response with developmental plasticity across diverse plant lineages (**Figure 7H**).

Our study demonstrates that *PutFER1*, a FER homolog in the halophyte *P. tenuiflora*, is transcriptionally upregulated under NaCl treatment and localizes to the plasma membrane, consistent with its reported function as a receptor kinase (Boisson-Dernier et al., 2011; Kessler et al., 2014). Functional validation in Arabidopsis revealed that *PutFER1* overexpression enhances root growth under salt stress and reduces ROS accumulation, accompanied by elevated activities of key antioxidant enzymes (SOD, POD, CAT, and APX). These results suggest that *PutFER1* contributes to salt tolerance by enhancing the ROS-scavenging capacity, thereby mitigating oxidative damage, which could be a critical mechanism that may be particularly critical in halophytes thriving in high-salinity environments.

In summary, our findings establish PutFER1 as a key regulator of salt tolerance through the modulation of ROS homeostasis, offering valuable insights into halophyte adaptation and presenting a promising genetic resource for breeding salt-resilient crops. However, the present study relied on heterologous expression in Arabidopsis, which may not fully recapitulate the native regulatory context of *P. tenuiflora*. Future work should include functional characterization of *PutFER1* in alkaligrass itself, using CRISPR-Cas9-mediated knockout or knockdown lines, to validate its role in a halophyte background (Zhang et al., 2021b). In addition, the precise signaling cascade downstream of *PutFER1*, including its potential ligands, co-receptors, and phosphorylation targets, remains to be elucidated in alkaligrass. Identifying interaction partners through methods such as co-immunoprecipitation followed by mass spectrometry would provide deeper mechanistic insights. Finally, field trials using *PutFER1*-overexpressing crop plants could assess its practical potential for improving salinity tolerance in agriculturally important species.

## Data Availability

All relevant data is contained within the article: The original contributions presented in the study are included in the article/**Supplementary Material**, further inquiries can be directed to the corresponding author/s.
