# Supplementary figures and images for: Genome-wide analysis of the CrRLK1L gene family in Puccinellia tenuiflora and functional study of PutFER1 in Arabidopsis underpinning salt tolerance
Source: Front Plant Sci. 2025 Nov 26;16:1680452. doi: 10.3389/fpls.2025.1680452 (PMC12689995; doi:10.3389/fpls.2025.1680452)

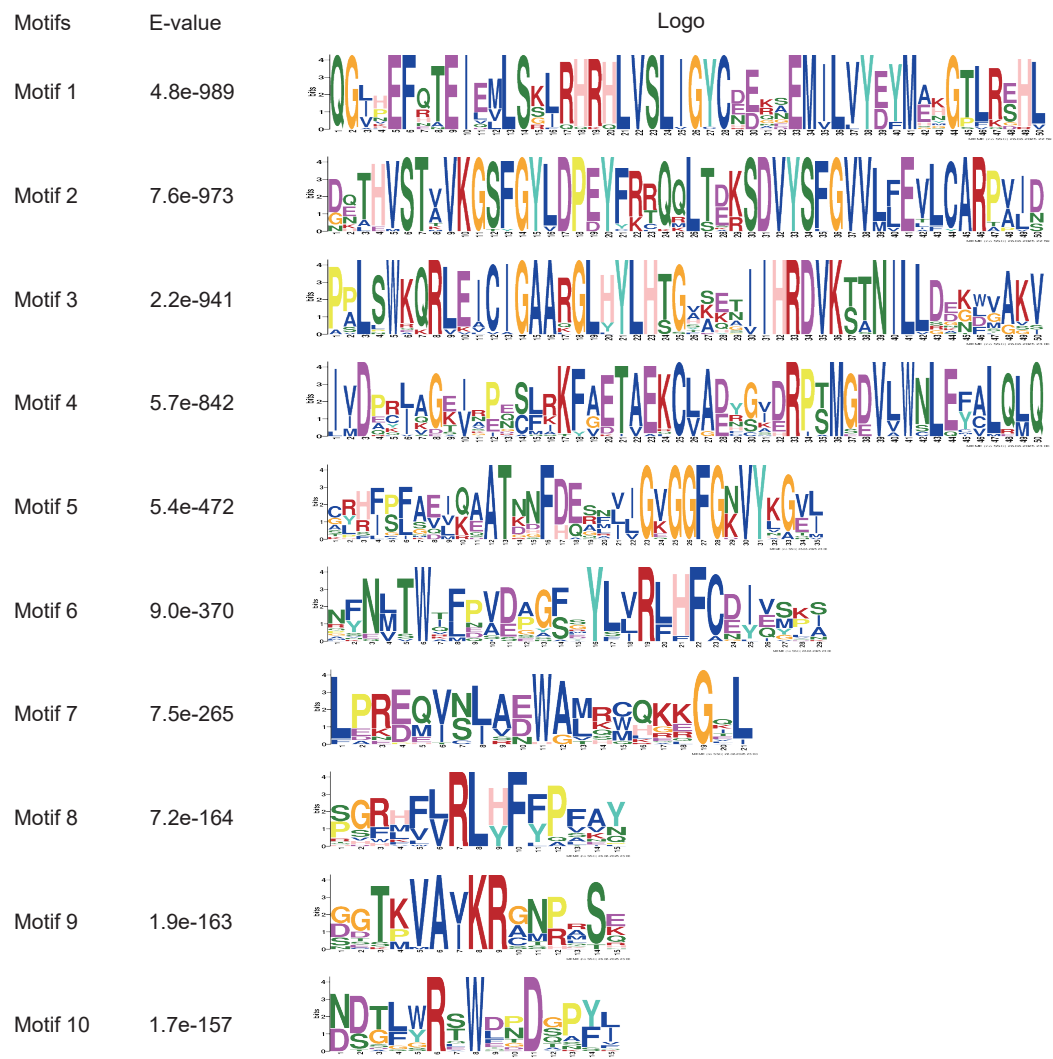

**Supplementary Figure 1. The logos showing the conserved residues in ten motifs of 25 *PutCrRLK1Ls*.**

Supplement: Supplementary file 1 [file DataSheet1.pdf]
