# Supplementary material for: Genome-wide analysis of the CrRLK1L gene family in Puccinellia tenuiflora and functional study of PutFER1 in Arabidopsis underpinning salt tolerance
Source: Front Plant Sci. 2025 Nov 26;16:1680452. doi: 10.3389/fpls.2025.1680452 (PMC12689995; doi:10.3389/fpls.2025.1680452)
Supplement: Supplementary file 2 [file DataSheet2.pdf]

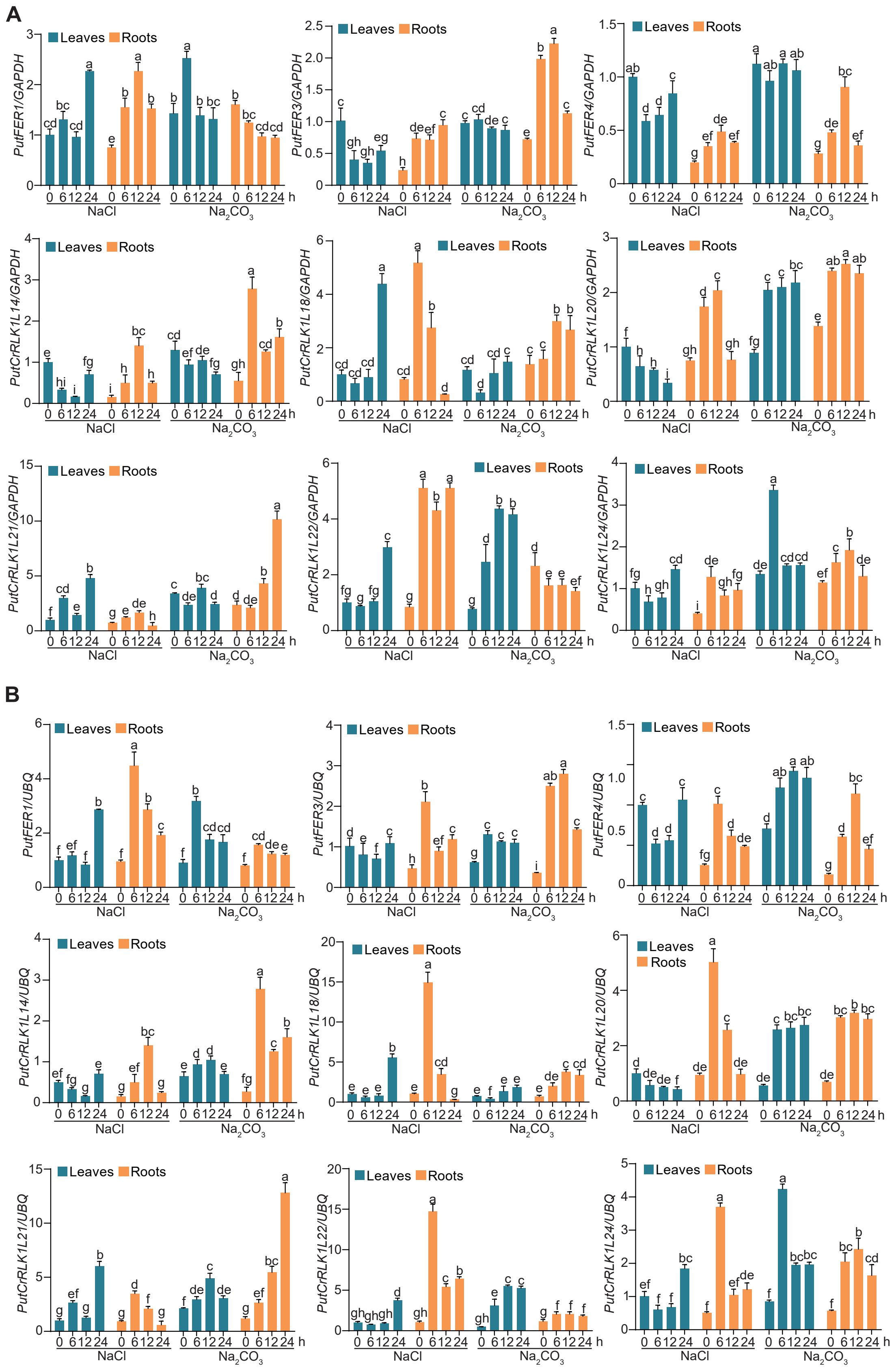

Supplementary Figure 2. Expression levels of *PutCrRLK1Ls* in leaves and roots using RT-qPCR analysis under Salt and alkali stress conditions.

The RT-qPCR results were calculated using the $2^{-\Delta\Delta Ct}$ method, with *PutGAPDH* (**A**) and *PutUBQ* (**B**) serving as reference genes for normalization. Different letters in (**A**) and (**B**) denote statistically significant differences (*P* < 0.01, one-way ANOVA). The expression levels of *PutCrRLK1Ls* were analyzed in 14-day-old alkaligrass seedlings treated with 200 mM NaCl or 100 mM $NaHCO_3$ (pH 9.0) for the indicated durations.
