# Supplementary material for: Genome-wide analysis of the CrRLK1L gene family in Puccinellia tenuiflora and functional study of PutFER1 in Arabidopsis underpinning salt tolerance
Source: Front Plant Sci. 2025 Nov 26;16:1680452. doi: 10.3389/fpls.2025.1680452 (PMC12689995; doi:10.3389/fpls.2025.1680452)
Supplement: Supplementary file 3 [file DataSheet3.pdf]

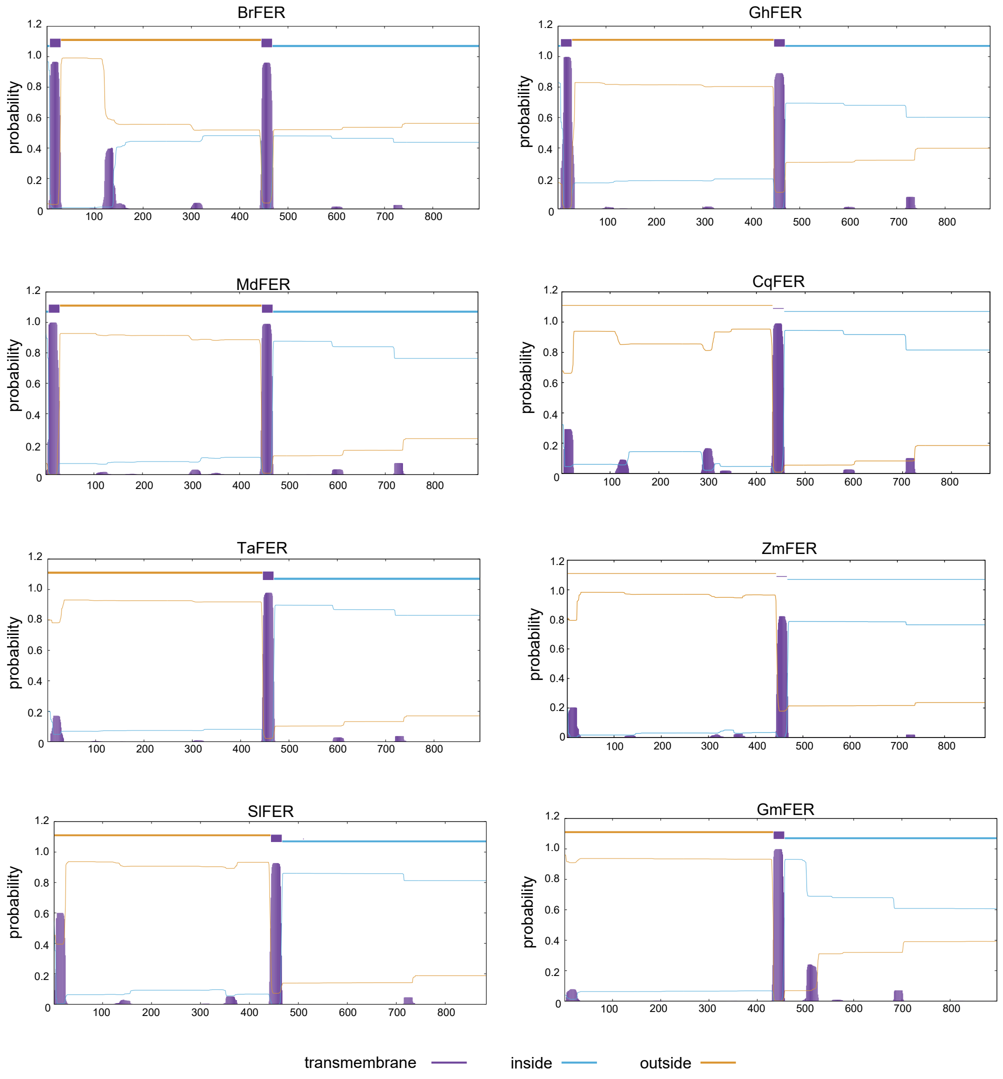

Supplementary Figure 3. Prediction of the transmembrane domain in FERs from various plant species. *Br*, *Brassica rapa*; *Cq, Chenopodium quinoa*; *Gh*, *Gossypium hirsutum*; *Gm*, *Glycine max*; *Md*, *Malus domestica*; *Sl*, *Solanum lycopersicum*; *Ta*, *Triticum aestivum*; *Zm*, *Zea mays*.
