# Supplementary material for: Genome-wide analysis of the CrRLK1L gene family in Puccinellia tenuiflora and functional study of PutFER1 in Arabidopsis underpinning salt tolerance
Source: Front Plant Sci. 2025 Nov 26;16:1680452. doi: 10.3389/fpls.2025.1680452 (PMC12689995; doi:10.3389/fpls.2025.1680452)
Supplement: Supplementary file 4 [file DataSheet4.pdf]

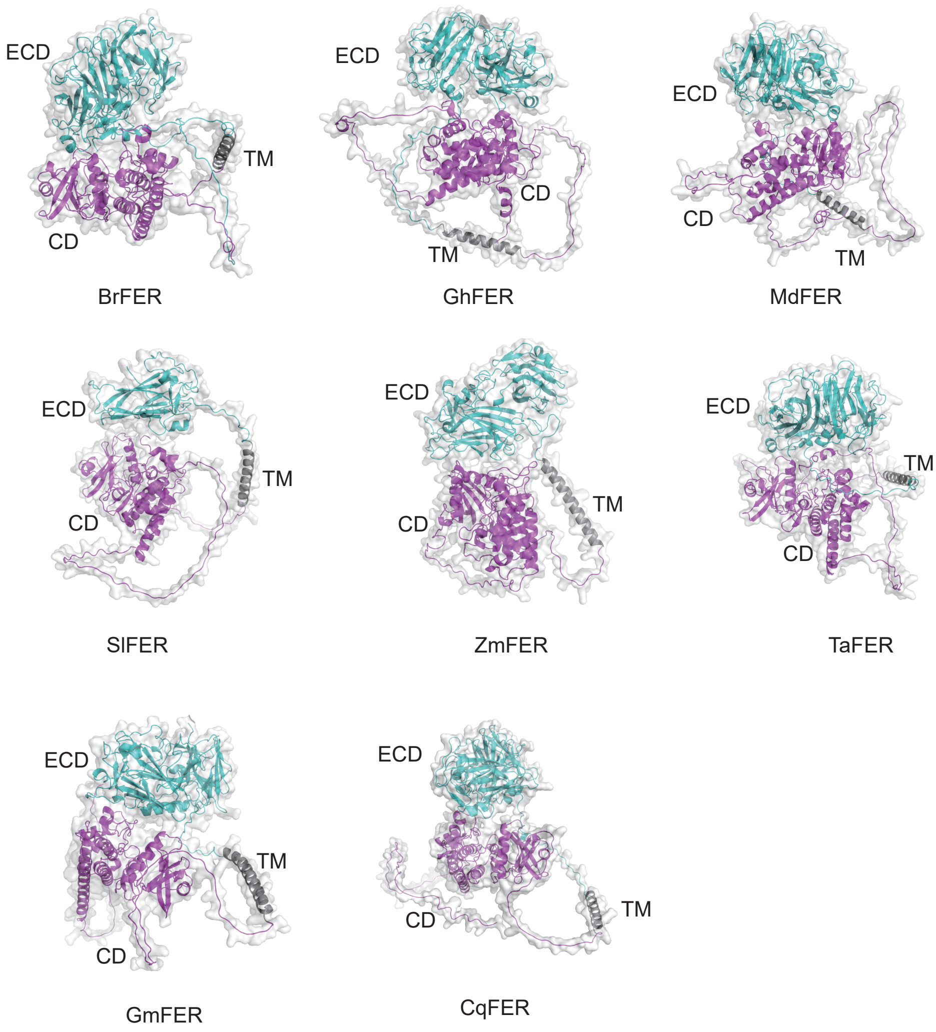

Supplementary Figure 4. Predicted 3D protein structure of FERs in various species.
Br, *Brassica rapa*; *CD*, *cytoplasmic domain*; *ECD*, *extracellular domain*; *Gh*, *Gossypium hirsutum*; *Gm*, *Glycine max*; *Md*, *Malus domestica*; *Sl*, *Solanum lycopersicum*; *Ta*, *Triticum aestivum*; *TM*, *transmembrane*; *Zm*, *Zea mays*.
