# Supplementary material for: Genome-wide analysis of the CrRLK1L gene family in Puccinellia tenuiflora and functional study of PutFER1 in Arabidopsis underpinning salt tolerance
Source: Front Plant Sci. 2025 Nov 26;16:1680452. doi: 10.3389/fpls.2025.1680452 (PMC12689995; doi:10.3389/fpls.2025.1680452)
Supplement: Supplementary file 5 [file DataSheet5.pdf]

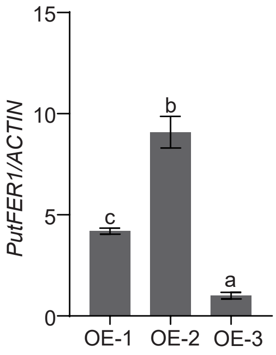

Supplementary Figure 5. Analysis of PutFER1 over-expression in heterologous transgenic Arabidopsis lines.
The RT-qPCR results were calculated using the $2^{-\Delta\Delta Ct}$ method, with *PutACTIN* serving as reference genes for normalization. Different letters denote statistically significant differences (P < 0.01, one-way ANOVA).
